# Predictive validity of a novel non-invasive estimation of effective shunt fraction in critically ill patients

**DOI:** 10.1186/s40635-019-0262-1

**Published:** 2019-08-20

**Authors:** Emma M. Chang, Andrew Bretherick, Gordon B. Drummond, J Kenneth Baillie

**Affiliations:** 1 Anaesthesia, Critical Care and Pain Medicine, Royal Infirmary of Edinburgh, Edinburgh, EH16 4SA UK; 2 MRC Institute of Genetics and Molecular Medicine, The University of Edinburgh, Edinburgh, EH4 2XU UK; 3 The Roslin Institute and Royal (Dick) School of Veterinary Studies, University of Edinburgh, Easter Bush, Edinburgh, EH25 9RG UK

**Keywords:** Oxygen, Shunt fraction, Arterial blood gas, Respiratory failure

## Abstract

**Background:**

Accurate measurement of pulmonary oxygenation is important for classification of disease severity and quantification of outcomes in clinical studies. Currently, tension-based methods such as P/F ratio are in widespread use, but are known to be less accurate than content-based methods. However, content-based methods require invasive measurements or sophisticated equipment that are rarely used in clinical practice. We devised two new methods to infer shunt fraction from a single arterial blood gas sample: (1) a non-invasive effective shunt (ES) fraction calculated using a rearrangement of the indirect Fick equation, standard constants, and a procedural inversion of the relationship between content and tension and (2) inferred values from a database of outputs from an integrated mathematical model of gas exchange (DB). We compared the predictive validity—the accuracy of predictions of P_a_O_2_ following changes in F_I_O_2_—of each measure in a retrospective database of 78,159 arterial blood gas (ABG) results from critically ill patients.

**Results:**

In a formal test set comprising 9,635 pairs of ABGs, the median absolute error (MAE) values for the four measures were as follows: alveolar-arterial difference, 7.30 kPa; P_a_O_2_/F_I_O_2_ ratio, 2.41 kPa; DB, 2.13 kPa; and ES, 1.88 kPa. ES performed significantly better than other measures (*p* < 10-10 in all comparisons). Further exploration of the DB method demonstrated that obtaining two blood gas measurements at different F_I_O_2_ provides a more precise description of pulmonary oxygenation.

**Conclusions:**

Effective shunt can be calculated using a computationally efficient procedure using routinely collected arterial blood gas data and has better predictive validity than other analytic methods. For practical assessment of oxygenation in clinical research, ES should be used in preference to other indices. ES can be calculated at http://baillielab.net/es.

**Electronic supplementary material:**

The online version of this article (10.1186/s40635-019-0262-1) contains supplementary material, which is available to authorized users.

## Background

Hypoxia is the defining feature of respiratory failure. Accurate quantification of pulmonary oxygenation defect is essential to determine inclusion in clinical trials, to measure outcomes in research studies, and to observe changes in lung function in a clinical setting.

In severely hypoxic patients, direct measurement of intrapulmonary shunt provides the most accurate quantification of an oxygenation defect [1]. Tension-based indices, including P_a_O_2_/F_I_O_2_ (P/F) ratio and alveolar-arterial (A-a) difference have poor agreement with intrapulmonary shunt fraction [1–3]. The primary limitation in tension-based indices is the marked and non-linear change in P_a_O_2_ when F_I_O_2_ is changed [4]. Brochard and colleagues demonstrated that this can be predicted from a simple mathematical model [5].

The concept of predictive validity is a mathematical reality check for a clinical measure. For a given clinical measure, predictive validity quantifies the extent to which that measure predicts an unseen event. The intent is not to predict the future, but rather to provide a rigourous, unbiased test of how well a clinical measure is describing a real entity: the assumption is that whichever measure is closest to the truth should also provide the best prediction. This approach, using mortality as the predicted event, was used in the development of consensus definitions for both acute respiratory distress syndrome (ARDS) [6] and sepsis [7].

A measure that accurately reflects the true state of a patient’s lungs should not change markedly following a change in F_I_O_2_. Therefore, the prediction of a P_a_O_2_ following a change in F_I_O_2_, assuming that the measure of the oxygenation defect remains unaltered, is a valid assessment for predictive validity.

We hypothesised that an easily understood, content-based oxygenation index may be obtainable from routinely-acquired arterial blood gas (ABG) data, without any need for additional invasive measurements. In order to assess different approaches, we quantified the predictive validity of P/F, A-a, and two new methods of estimating shunt fraction (effective shunt fraction (ES) and a database method (DB)) in a simple test: prediction of P_a_O_2_ following a change in F_I_O_2_ in a large retrospective cohort.

## Methods

### Data source and filtering

We used a set of 78,159 arterial blood gas samples taken between 2011 and 2016 from 6511 patients on the general intensive care unit (ICU) at the Royal Infirmary of Edinburgh. The unit admits adult patients, with predominantly emergency medical, trauma, and general surgery conditions—not elective cardiac or thoracic surgery or neurosurgery. We did not study patients who had ECMO. The samples were routine analyses: the F_I_O_2_ value was input by the clinician performing the analysis. The analysis machine was maintained by the clinical chemistry department and regularly calibrated against known standards.

To obtain sample sets in which underlying pulmonary pathology was unlikely to change substantially between samples, we limited the selection of samples to pairs of ABGs that met the following inclusion criteria: (1) taken within a 3-h window, (2) taken from a mechanically ventilated patient, (3) where the F_I_O_2_ was reduced between the first and the second sample, and (4) where alveolar ventilation was stable (change in P_a_CO_2_ < 0.3 kPa).

### Derivation of effective shunt fraction

ES expresses the shunt fraction that would be required to produce a given impairment in oxygenation, that is the proportion of cardiac output that would have to shunt in order to have this effect (i.e. to produce this degree of hypoxia). In clinical practice, it will almost never be the case that a given patient has pure shunt; ES is intended to provide an intuitive and consistent quantification of oxygenation impairment.

Full details of the methods used are given in the Additional file 1. Briefly, ES was first calculated from the blood gas results as follows.

The shunt equation is usually expressed in the following way [8]:

1 $$ \frac{Q_{S}}{Q_{T}} = \frac{C_{c'}O_{2} - C_{a}O_{2}}{C_{c'}O_{2} - C_{v}O_{2}}  $$

All of the necessary variables can be easily calculated from routine clinical measurements, with the exception of C_v_O_2_. We applied the Fick principle for oxygen uptake (see Additional file 2), in order to replace this term:

2 $$ \frac{Q_{S}}{Q_{T}} = \frac{C_{c'}O_{2} - C_{a}O_{2}}{C_{c'}O_{2} - C_{a}O_{2} - \frac{VO_{2}}{Q}}  $$

After estimating P_A_O_2_ using the alveolar gas equation, arterial (C_a_O_2_) and end-capillary (C_c’_O_2_) oxygen contents were derived using model equations from Dash and Bassingthwaighte [9], using measured pH and P_a_CO_2_ to estimate the PO_2_ at which Hb is 50% saturated (P_50_). Values for oxygen consumption (VO_2_) and cardiac output (Q) were set at single values in the physiological range (see Additional file 1).

### Predictive validity

Each measure was quantified for the earlier ABG in each pair. For ES, P/F and A-a, the F_I_O_2_ and P_A_O_2_ for the second ABG was used in the rearranged equations (derived in Additional file 1) to estimate the new P_a_O_2_, using the same value for the oxygenation index under inspection.

For measurement of predictive validity in the ES method, a predicted C_a_O_2_, after change of F_I_O_2_, was calculated using F_I_O_2_ and P_A_O_2_ from the second ABG:

3 $$ C_{a}O_{2} = C_{c'}O_{2} - \frac{Q_{S}/Q_{T} \times VO_{2}}{Q_{T} - Q_{S}}  $$

This value of C_a_O_2_ was then converted to a predicted P_a_O_2_ value using the method of Dash and Bassingthwaighte [9].

**Fig. 1:**
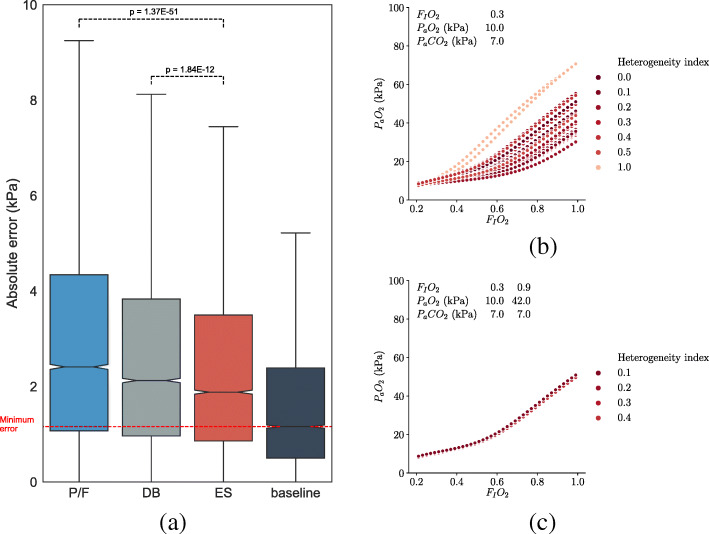
**a** Boxplot showing distribution of absolute error for each measure in all samples, together with baseline distribution of pairs of ABGs in which F_I_O_2_ was unchanged (box shows mean +/ − one quartile, whiskers show range). **b** Range of possible F_I_O_2_-P_a_O_2_ combinations for conditions matching a single ABG result. **c** Range of possible results for conditions matching two ABG results at different F_I_O_2_

In order to minimise the noise generated (affecting all measures) by changes in ventilation or circulation, we focused our study on patients whose F_I_O_2_ was being weaned downwards. The median absolute differences between predicted and observed P_a_O_2_ across all ABG pairs were taken as the predictive validity for each measure. For the DB method, input settings for an integrated mathematical model of gas exchange were identified which matched to the first ABG. These were then extrapolated to the F_I_O_2_ and P_a_CO_2_ of the second ABG, and the mean P_a_O_2_ of all matching model runs taken to be the prediction (Fig. 1b). Further details of the mathematical model used for the DB method can be found in supplementary content (see Additional file 2).

### Research Ethics

Ethical approval was obtained from the Scotland A Research Ethics Committee [16/SS/0209].

### Software and statistical analyses

All analysis was performed using Python 3.5.2 and scipy.stats version 0.18.1. A Kruskal-Wallis *H* test was used to determine the difference in error rate between the different measures. Mann-Whitney *U* tests with Bonferroni correction were used as a stringent post test for pairwise comparisons.

## Results

### Comparison of oxygenation measures

From the total set of 78,159 ABGs from 6511 patients, an initial test set was selected at random, containing 54,115 ABGs from 4558 patients. From this random sample, we selected a formal test set comprising 9635 pairs of ABGs, which met the criteria listed above, pairs of ABGs taken from mechanically ventilated patients within a 3-h window, where the F_I_O_2_ was reduced between the first and the second sample, and where alveolar ventilation was stable. When we compared the predicted with the measured P_a_O_2_ values in the second values of these pairs, the median absolute error (MAE) values for the four measures considered were as follows: A-a, 7.30 kPa; P/F, 2.41 kPa; DB, 2.13 kPa; and ES, 1.88 kPa. ES had significantly superior predictive validity than all other measures (Table 1).

**Table 1:** Pairwise comparisons between errors in oxygenation measures in test set (Mann-Whitney *U* test, Bonferroni correction)

Measure 1	Measure 2	*p* (MWu)
A-a	P/F	< 1E −300
A-a	DB	< 1E −300
A-a	ES	< 1E −300
P/F	DB	6.86E −17
P/F	ES	1.37E −51
DB	ES	1.84E −12

Effective shunt values in this population ranged from 0 to 63% (mean 16.1%, SD 8.6%). P/F values (kPa) ranged from 6.65 to 84.7 (mean 32.2, SD 12.5). A-a values (kPa) ranged from 0 to 81.2 (mean 22.7, SD 13.6).

### Validation of assumed values

Three key assumed values are required for the calculation of ES: respiratory exchange ratio (RER), cardiac output (Q), and metabolic oxygen consumption (VO_2_). In order to prevent bias, we optimised estimation of these assumed parameters in a training set comprising 30% of the available ABGs (*n*=24,044), selected at random. Varying the three values over wide ranges (RER, 0.8 to 1.1; Q, 3 to 15 l.min^−1^; VO_2_, 0.15 to 1 l.min^−1^) had minimal effect on the value of effective shunt.

### Multiple ABGs under different conditions

Although the DB method does not perform as well as the simpler and less computationally demanding ES method, it provides an opportunity to test the effect of obtaining multiple ABGs at different F_I_O_2_. The model takes standard physiological inputs, including pure shunt fraction, V/Q heterogeneity index, cardiac output, and F_I_O_2_ and returns blood gas results at steady state. The database of model results enables us to infer the possible physiological conditions that could give rise to a given ABG result. With one ABG, a wide range of possibilities remain (Fig. 1b); a second ABG at a different F_I_O_2_ substantially constrains the range of possible physiological states that describe a given patient (Fig. 1c).

## Discussion

This is the first study, to our knowledge, comparing the predictive validity of non-invasive effective shunt fraction with tension-based measures. Our observations are consistent in magnitude and direction with previous work studying changes in measures of oxygenation in human participants [1–3].

The poor performance of A-a difference is consistent with the report by Cane and colleagues [1], who demonstrated that A-a was the least reliable measure compared with invasive measurements of Qs/Qt. The very low predictive validity of A-a in our study, together with previous work, leads us to conclude that this measure has no role in any context.

Our study has several limitations. The ABG data itself was obtained from electronic records whereby F_I_O_2_ was entered by the treating clinician (nurse/doctor/nurse practitioner), which is a possible source of error. Importantly, this potential error applies equally to all measures of oxygenation.

To compare the integrity of each oxygenation measure alone, we assumed that baseline physiological function is not altered by the change of F_I_O_2_. However, increases in F_I_O_2_ cause absorption atelectasis [10]. We have mitigated this by restricting our analysis to pairs of ABGs in which the F_I_O_2_ was decreasing. Since the reversal of absorption atelectasis is slower than the onset [10], and there are fewer sudden changes in oxygenation in this group, we expect that restricting our analysis to weaning patients will mitigate this source of noise.

Marked changes in P_a_O_2_ may occur within a 3-h interval due to real changes in pulmonary function, for example due to recruitment, suction, diuresis, or change in posture. We therefore cannot draw any inference from the absolute value of the error in prediction, only a comparison between different methods. Noise caused by these and other factors is expected to limit the maximum possible accuracy of any prediction of P_a_O_2_. This minimum achievable error is reflected by the baseline values (Fig. 1a) showing the change in P_a_O_2_ between pairs of ABGs meeting the other selection criteria, with no change in F_I_O_2_.

There is also a significant limitation in the concept of reducing the full complexity of pulmonary oxygenation to a single numerical value. All clinical measurements are subject to this limitation—they provide summary measurements that require informed interpretation. The lung is no different from any other system in this regard. Studies using the multiple gas elimination technique (MIGET) have confirmed that lung injury leads to substantial heterogeneity in the matching of ventilation to perfusion, which causes hypoxia without pure shunt [11, 12]. ES, by design, combines these mechanisms into a single value from a three-compartment model: the amount of pure shunt that would be needed to have a given effect on oxygenation.

Since V/Q heterogeneity and shunt are separate inputs into the physiological model used to generate the database (see Additional file 1), the database approach is expected to handle this distinction better than the other measures. However, as shown in Fig. 1b, there is insufficient information in a single ABG to distinguish between shunt and V/Q heterogeneity. In contrast, with two ABGs taken at different settings of F_I_O_2_, the patient’s oxygen responsiveness is quantified, greatly restricting the range of possible values for both shunt and V/Q heterogeneity (Fig. 1c). This double F_I_O_2_ test may resolve the uncertainty in quantifying pulmonary shunt but is, at present, computationally demanding.

The striking superiority of ES in the context of critical illness may lead to an increase in clinical use. We support this, in part because the value itself is intuitive to critical care clinicians and is comparable across different health care systems and measurement units. Although it performs substantially better than other measures, it should be noted that ES is an imperfect measure and is not expected to be completely independent of extra-pulmonary factors, including F_I_O_2_, alveolar ventilation, and intracardiac shunt.

## Conclusions

The effective shunt fraction, a new, non-invasive method of estimating shunt, can be calculated on any ABG result, provided the F_I_O_2_ is known. The computation is fast and simple. Hence, the method could be retrospectively applied to previous studies that hold ABG data in a machine-readable format. Whilst the simplicity of the P/F ratio will continue to make it a popular choice for clinical use, the superior predictive validity of ES makes it a better choice where accurate quantification of oxygenation defect is necessary.

## Additional files

Additional file 1 Supplementary information. Includes the derivation of oxygenation measures and method of predicting P_a_O_2_, as well as optimisation of assumed variables using the test dataset. (PDF 227 kb)

Additional file 2 Integrated model of gas exchange. Structure of an integrated computational model of oxygen delivery which was used to generate a database of model results for inferring the possible physiological conditions that could give to rise to any given ABG result (“DB” method in the main manuscript). (PDF 80 kb)

## Data Availability

An online calculator to compute the effective shunt fraction is available at: http://baillielab.net/es Python code to calculate the effective shunt fraction is available from github: http://github.com/baillielab

## Abbreviations

A-a: Alveolar-arterial difference
ABG: Arterial blood gas
ARDS: Acute respiratory distress syndrome
C_a_O_2_: Arterial blood oxygen content
C_c’_O_2_: End-capillary blood oxygen content
DB: Database method
ES: Effective shunt
F_I_O_2_; ICU: Intensive care unit
MWu: Mann-Whitney *U* test
P/F: The ratio of P_a_O_2_ in the arterial blood to the therapeutic MAE: Median absolute error
Q: Cardiac output
Qs/Qt: Shunt fraction
RER: Respiratory exchange ratio
V/Q: Ventilation/perfusion ratio
VO_2_: Metabolic oxygen consumption
