# Correction to: Predictive validity of a novel non-invasive estimation of effective shunt fraction in critically ill patients

**DOI:** 10.1186/s40635-020-00320-4

**Published:** 2020-07-10

**Authors:** Emma M. Chang, Andrew Bretherick, Gordon B. Drummond, J. Kenneth Baillie

**Affiliations:** 1 Anaesthesia, Critical Care and Pain Medicine, Royal Infirmary of Edinburgh, Edinburgh, EH16 4SA UK; 2 MRC Institute of Genetics and Molecular Medicine, The University of Edinburgh, Edinburgh, EH4 2XU UK; 3 The Roslin Institute and Royal (Dick) School of Veterinary Studies, University of Edinburgh, Easter Bush, Edinburgh, EH25 9RG UK

**Correction to: ICMx 7, 49 (2019)**

**https://doi.org/10.1186/s40635-019-0262-1**

Following publication of the original article [1], the authors reported an error in Eq. 2 of the article; the article is missing brackets.

Please find the correct version (with the brackets added) of the equation in this correction.

The authors apologize for any inconvenience caused.

$$ \frac{Q_S}{Q_T}=\frac{C_{c\hbox{'}}{O}_2-{C}_a{O}_2}{C_{c\hbox{'}}{O}_2-\left({C}_a{O}_2-\frac{VO_2}{Q}\right)} $$
