# Supplementary material for: Predictive validity of a novel non-invasive estimation of effective shunt fraction in critically ill patients
Source: Intensive Care Med Exp. 2019 Aug 20;7:49. doi: 10.1186/s40635-019-0262-1 (PMC6701711; doi:10.1186/s40635-019-0262-1)
Supplement: Supplementary file 1 — Supplementary information. Includes the derivation of oxygenation measures and method of predicting PaO2, as well as optimisation of assumed variables using the test dataset. (PDF 227 kb) [file 40635_2019_262_MOESM1_ESM.pdf]

Supplementary information

Derivation of oxygenation measures and prediction of P_aO_2

Estimated Shunt Fraction

Estimated shunt fraction was calculated by assuming the necessary values to complete the shunt equation:

$$\frac{Q_S}{Q_T} = \frac{C_{c'}O_2 - C_aO_2}{C_{c'}O_2 - C_vO_2} \tag{1}$$

An estimation of the shunt fraction (Qs/Qt) was calculated using a combination of Equation 1 and the direct Fick equation to form Equation 2. Arterial (C_aO_2) and end-capillary ($C_{c'}O_2$) oxygen contents were derived using model equations from Dash and Bassingthwaighte.

Combining this value with P_aO_2 allows C_aO_2 to be obtained. $C_{c'}O_2$ was estimated using P_AO_2 from the alveolar gas equation (Equation 9). The combination of the two equations on the left provides Equation 10 from which we obtained C_vO_2.

Values for oxygen consumption (VO_2) and cardiac output (Q) were set at single optimal values in the physiolgical range (see below).

$$C_vO_2 = C_aO_2 \times \frac{DO_2 - VO_2}{DO_2}$$

$$DO2 = C_aO_2 * Q$$

$$C_vO_2 = C_aO_2 - \frac{VO_2}{Q}$$

$$\frac{Q_S}{Q_T} = \frac{C_{c'}O_2 - C_aO_2}{C_{c'}O_2 - C_aO_2 - \frac{VO_2}{Q}} \tag{2}$$

By utilizing an expanded version of Equation 1 the following expression was formed to estimate the 'new' C__aO_2 in ABG2:

$$C_aO_2 = C_{c'}O_2 - \frac{Q_S/Q_T \times VO_2}{Q_T - Q_S} \tag{3}$$

Derivation of the Estimated Shunt equation from the expanded shunt fraction:

$$Q_S(C_{c'}O_2 - C_vO_2) = Q_T(C_{c'}O_2 - C_aO_2)$$

$$Q_TC_aO_2 = Q_TC_{c'}O_2 - Q_SC_{c'}O_2 + Q_SC_vO_2$$

$$Q_TC_aO_2 = Q_TC_{c'}O_2 - Q_SC_{c'}O_2 + Q_S\left(\frac{Q_TC_aO_2 - VO_2}{Q_T}\right)$$

$$Q_TC_aO_2 = Q_TC_{c'}O_2 - Q_SC_{c'}O_2 + Q_SC_aO_2 - \frac{Q_SVO_2}{Q_T}$$

$$(Q_T - Q_S)C_aO_2 = (Q_T - Q_S)C_{c'}O_2 - (Q_S/Q_T \times VO_2)$$

$$C_aO_2 = C_{c'}O_2 - \frac{Q_S/Q_T \times VO_2}{Q_T - Q_S} \tag{4}$$

The corrected version of Dash and Bassingthwaighte's work [1] provides simple mathematical expressions based on the nonlinear biochemical interactions of O_2 and CO_2 with Hb. The invertible equation contains 'binding constants' (KHb_{O2}) that depend on P_aO_2 among other factors. By reversing the equation, our P_{50} function can predict P_aO_2 from Hb saturation and vice-versa.

An online calculator to compute the effective shunt fraction is available at:

http://baillielab.net/es

Python code to calculate the effective shunt fraction is available from github:

http://github.com/baillielab

P/F Ratio

P/F was first calculated using the P_aO_2 and corresponding F_IO_2 from ABG1. By rearranging Equation 5 the new P_aO_2 can be predicted using the original P/F value with the new F_IO_2 value from ABG2 via Equation 6.

$$P/F = \frac{P_aO_2}{F_IO_2} \tag{5}$$

$$P_aO_2 = P/F \times F_IO_2 \tag{6}$$

Alveolar-arterial difference:

The equation for Alveolar-arterial difference is shown below:

$$Aa\,difference = P_AO_2 - P_aO_2 \tag{7}$$

Therefore Equation 8 was used to estimate P_aO_2 following a change in F_IO_2.

$$P_aO_2 = P_AO_2 - Aa\,difference \tag{8}$$

Whereby:

$$P_AO_2 = P_IO_2 - \frac{P_aCO_2}{RER} + \left[F_IO_2 \times P_aCO_2 \times \left(\frac{1 - RER}{RER}\right)\right] \tag{9}$$

Where:

$$DO_2 = Q \times C_aO_2$$

and

$$C_vO_2 = \frac{(DO_2 - VO_2)}{Q}$$

$$VO_2 = Q \times (C_aO_2 - C_vO_2) \tag{10}$$

Alveolar oxygen partial pressure (P_AO_2) was calculated using values from ABG1 to obtain the A-a difference, then calculated again with values from ABG2 to predict P_aO_2 in Equation 8. RER was kept constant at 0.8 for ES and A-a difference.

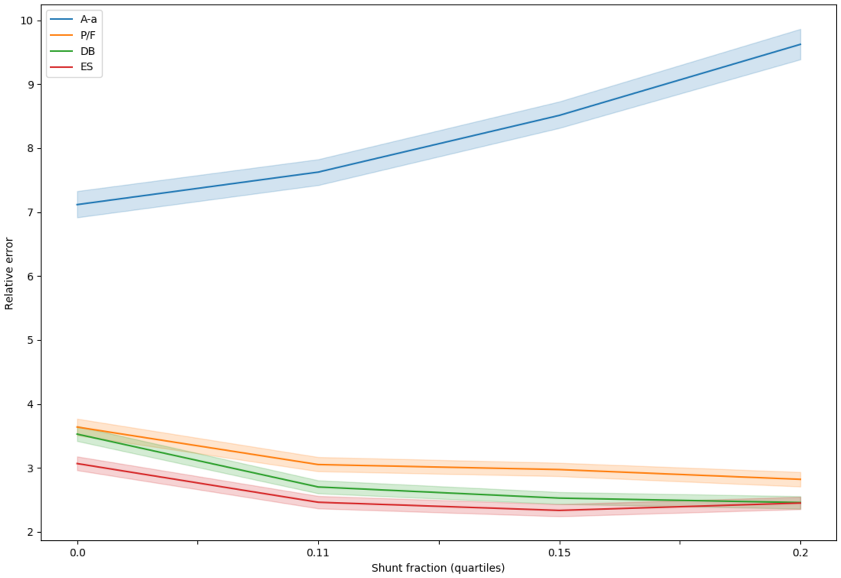

Figure 1: Change in relative error (absolute error / P_aO~2 in second ABG) for each measure across shunt severity quartiles.

Distribution of ES values

The distribution of ES values in the study population is shown in Figure 2.

Optimisation of assumed variables

The test set of ABGs was used for optimisation of the variables listed below. Although VO2 and Q have a direct impact on the value of ES (Equation 2), when these values are held constant they have no impact on the accuracy of predictions made using ES (Figure 4 and Figure 5).

Respiratory exchange ratio (RER)

Both the calculation of the $C_{c'}O_2$ term in Equation 2 for effective shunt fraction (ES) and the alveolar term in A-a difference require the alveolar gas equation, Equation 9, which uses RER to calculate P_AO_2. We therefore considered the possibility that changing the assumed RER would affect the predictive validity of these measures.

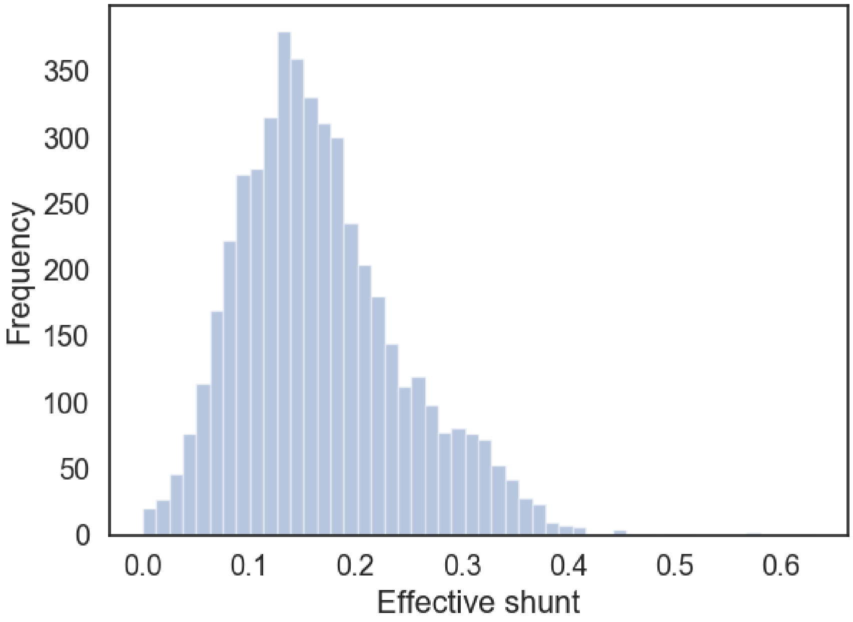

Figure 2: Histogram of calculated ES values for all members of the critically ill population in this study.

Varying the assumed RER across the range 0.7 to 1.2 had negligible effect on ES and a slight effect on A-a (Figure 3). We therefore chose a value of 1 for

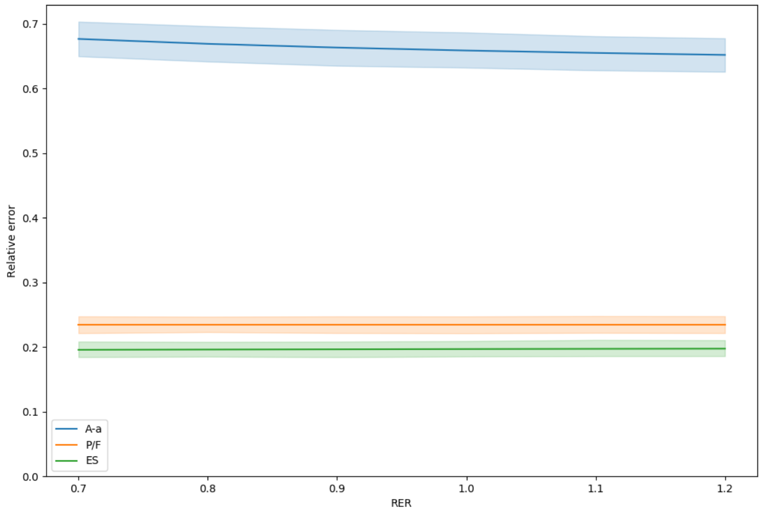

Figure 3: Change in relative error (absolute error / P_aO~2 in second ABG) of P/F, ES, and A-a across a range of assumed values for RER

Cardiac output (Q)

Changing cardiac output across the range 1 to 15 $l.min^{-1}$ had minimal effect on the accuracy of predictions by ES (Figure 4)

Metabolic oxygen consumption (VO2)

Likewise, a change in VO2 does not alter the predictive validity of ES.

Unselected patients

In order to limit noise in our predicitive validity, we selected weaning patients, in whom it is expected that there is less chance of unexpected changes in pulmonary pathophysiology between any pair of ABGs. Here, we report the results of the same analyses performed in an unselected group of patients (Figure 6). As expected with additional noise, the signals are weaker, but the direction of

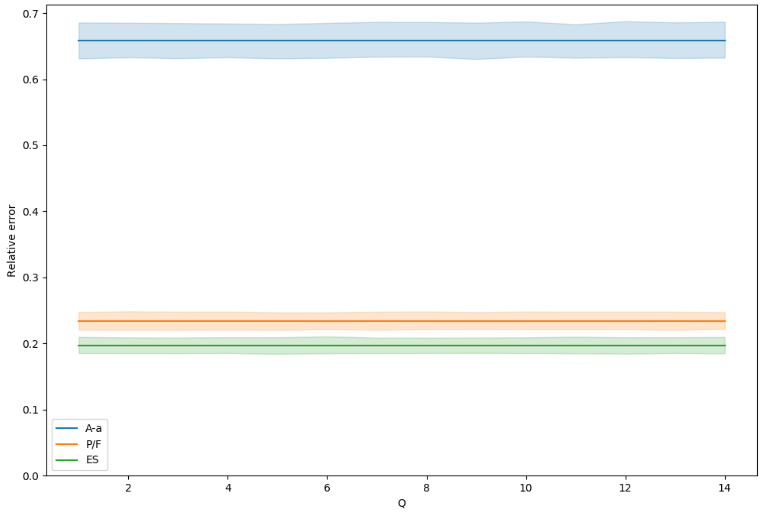

Figure 4: Change in relative error (absolute error / P_aO~2 in second ABG) of P/F, ES, and A-a across a range of assumed values for Q

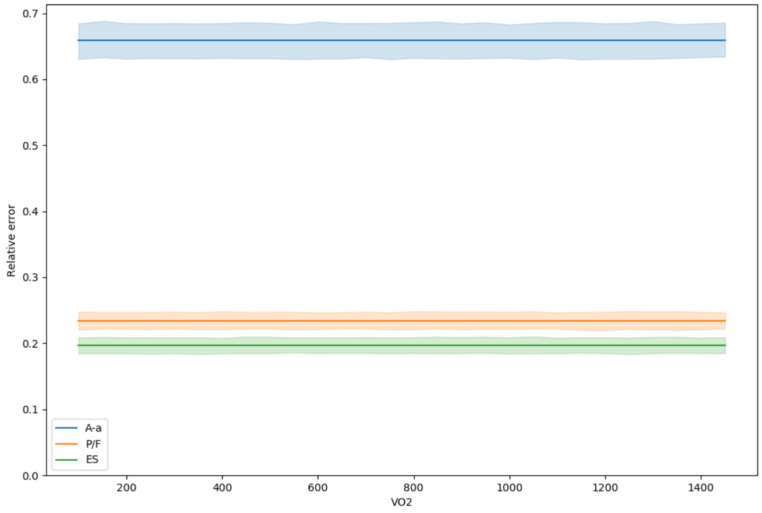

Figure 5: Change in relative error (absolute error / P_aO~2 in second ABG) of P/F, ES, and A-a across a range of assumed values for Q

effect and statistical significance remain robust (Table 1). Median differences from baseline were: A-a, 6.08kPa; P/F, 1.33kPa; DB, 1.31kPa; ES: 0.97kPa.

Table 1: Pairwise comparisons between errors in oxygenation measures in unselected patients (Mann-Whitney U test, Bonferroni correction).

Measure	Measure	p (MWu)	Threshold
A-a	P/F	$<$ 1E-300	0.008
A-a	DB	$<$ 1E-300	0.008
A-a	ES	$<$ 1E-300	0.008
P/F	DB	1.57E-01	0.008
P/F	ES	4.65E-17	0.008
DB	ES	3.47E-19	0.008

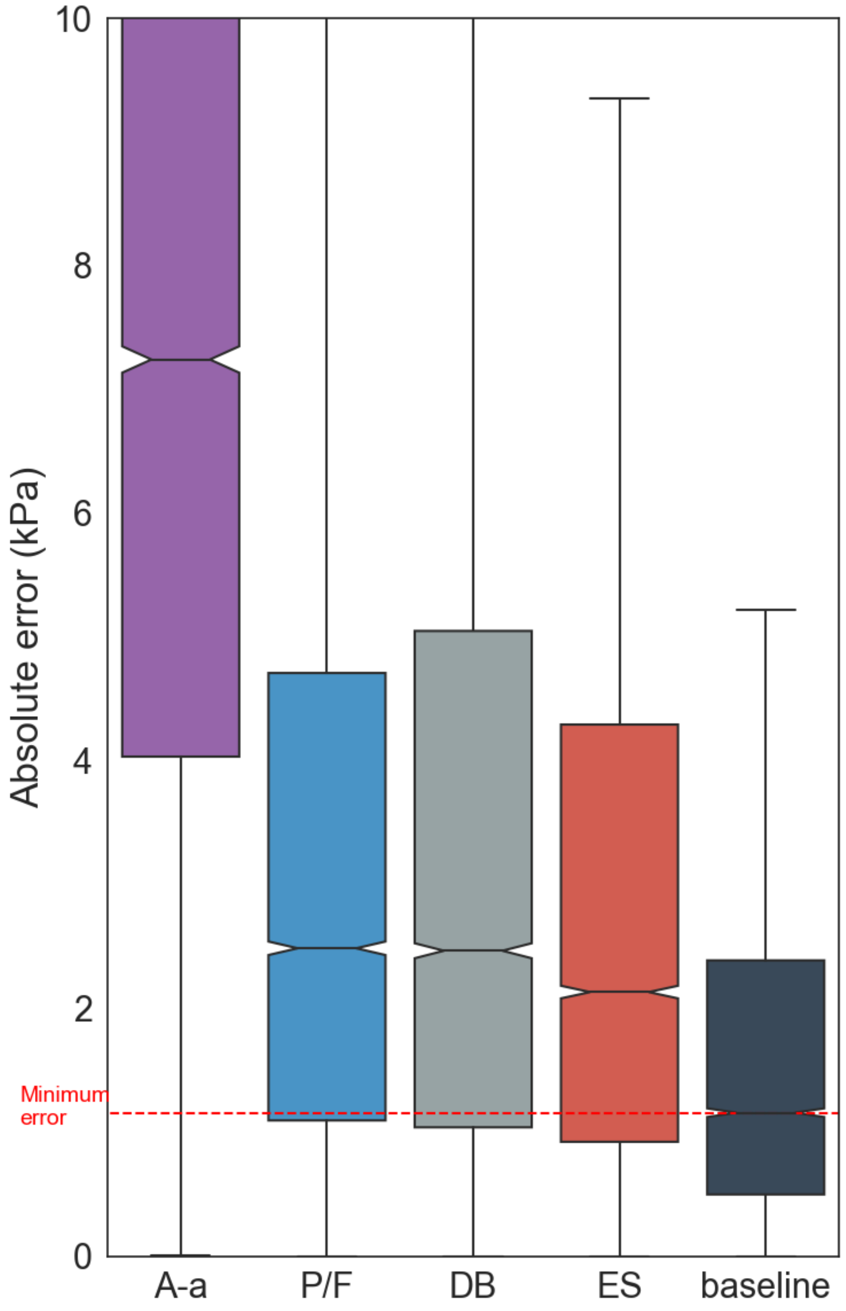

Figure 6: Boxplot showing distribution of absolute error for each measure in unselected samples, together with baseline distribution of pairs of ABGs in which F_IO_2 was unchanged (box shows mean +/- one quartile, whiskers show range).

References

1 Dash RK, Bassingthwaighte JB. Erratum to: Blood hbo2 and hbco2 dissociation curves at varied o2, co2, pH, 2,3-dpg and temperature levels. *Annals of biomedical engineering* 2010;**38**:1683–701. doi:10.1007/s10439-010-9948-y
